# Temporal Changes in Concentrations of Lead and Other Trace Metals in Free-Ranging Eurasian Eagle Owls *Bubo bubo* in Sweden

**DOI:** 10.1007/s00244-019-00654-5

**Published:** 2019-07-16

**Authors:** Björn Helander, Marcus Sundbom, Agneta A. Runkel, Anders Bignert

**Affiliations:** 1 Department of Environmental Research and Monitoring, Swedish Museum of Natural History, Box 50007, 104 05 Stockholm, Sweden; 2 Department of Environmental Science and Analytical Chemistry (ACES), Stockholm University, Stockholm, Sweden; 3 Environmental Sciences, Jožef Stefan Institute, Ljubljana, Slovenia

## Abstract

Patterns of lead and other trace metals were examined in 122 Eurasian eagle owls *Bubo bubo* found dead in Sweden in the period 1978–2013. Environmental lead (Pb) has decreased over recent decades from reduced anthropogenic emissions but mortality by Pb poisoning is still frequently reported for avian raptors and scavengers exposed to Pb ammunition. One objective here was to determine if Pb concentrations in a nocturnal non-scavenging raptor follow the general decline observed in other biota. Pb concentration in owl liver was significantly correlated with body weight, sex, latitude, longitude and season. Pb showed a significant decreasing trend towards north and west. Starved birds had significantly higher concentrations. Total Pb concentrations in liver averaged 0.179 μg g^−1^ dry weight (median 0.103) and decreased by 5.6% per year 1978–2013, or 5.3% after adjustment for confounding factors, similar to trends in other species. Among 14 other trace elements only antimony and arsenic showed decreasing trends. Lead isotope ratios ^206^Pb/^207^Pb and ^208^Pb/^207^Pb increased from 1.138 and 2.408 in 1978–1985 to 1.170 and 2.435 in 2010–2013, respectively, demonstrating that the decreasing Pb concentration in eagle owl is related to the phase-out of leaded gasoline in Europe, where Pb additives had much lower isotope ratios than natural lead in Swedish soils. Only one incidence of suspected Pb poisoning (40.7 μg g^−1^ in liver) was observed indicating that poisoning from ingestion of metallic lead is rare (< 1%) in eagle owl in Sweden, in contrast to what has been reported for eagles.

Lead (Pb) is a naturally occurring but nonessential element that is highly toxic at elevated concentrations and with a potential to affect most body systems in animals. Exposure to Pb and incidences of Pb poisoning have been extensively reported for several diurnal birds of prey around the world, notably eagles and vultures (Wayland and Bollinger 1999; Kurosawa 2000; Church et al. 2006; Pattee et al. 2006; Krone et al. 2009; Franson and Russel 2014; Berny et al. 2015; Mateo-Tomás et al. 2016; Ecke et al. 2017; Wiemeyer et al. 2017; Isomurso et al. 2018), and scarcely for the mainly nocturnal Eurasian eagle owl *Bubo bubo* (Mateo et al. 2003, Kim and Oh 2012) and great horned owl *Bubo virginianus* (Clark and Scheuhammer 2003). Mortality from Pb poisoning in birds is mostly associated with ingestion of lead-shot or bullet fragments of hunting ammunition and associated with feeding habits occurring mainly in various ducks and raptorial birds (summarized in Franson and Pain 2011). Effects from elevated but sublethal concentrations of Pb include inhibition of enzyme systems and immunosuppression (Franson and Pain 2011). Decreased delta-aminolevulinic acid dehydratase (δALAD) activity correlated with elevated lead concentrations in blood has been demonstrated experimentally with American kestrels (*Falco sparverius*) (Franson et al. 1983) and in eagle owl nestlings from a mining district in Spain (Gómez-Ramírez et al. 2011; Espín et al. 2015), and depressed cell-mediated immunity was recorded in an experimental study with red-tailed hawks (*Buteo jamaicensis*) (Redig et al. 1991). The large-sized eagle owl may risk higher exposure to contaminants, including Pb, than most other owls due to its potentially higher position in the food web. Eagle owls are known to feed on a wide variety of prey: mammals of sizes from voles to hares, birds of almost all kinds from medium-sized passerines to ducks and including other raptors, and even some fish and amphibians (summarized in Mikkola 1983; Cramp 1985). Notably important prey in Sweden are water vole (*Arvicola terrestris*), brown rat (*Rattus norvegicus*), hares (*Lepus* spp.), mallard (*Anas platyrhynchos*), coot (*Fulica atra*), and gulls (*Laridae*) but with considerable variations in occurrence in prey samples between biotopes (Höglund 1966; Olsson 1979). Thus, the eagle owl can be characterized as an opportunistic feeder with a varying composition of prey that reflects the availability in different habitats and seasons. The Eurasian eagle owl is not known to utilize carrion and should therefore not (or very rarely) be exposed to lead fragments from rifle ammunition in slaughter offal from hunting, or shotgun pellets in carcasses. Conversely, there also is a possibility that eagle owls would select some live prey with aberrant behavior and including victims of a gunshot wound. In white-tailed sea eagles (*Haliaeetus albicilla*), a facultative but near obligate carrion feeder in Sweden, concentrations of lead in liver did not decline from 1981 to 2004, in contrast to what has been observed in other biota, and it was concluded that the reason was ingestion of lead from ammunition (Helander et al. 2009). In this study, we focused on concentrations and temporal changes of lead and stable lead isotopes in banked liver tissue from free-ranging eagle owls found dead in Sweden more than 36 years. We hypothesized that ingestion of lead from ammunition by eagle owl is limited in Sweden and that Pb concentrations in eagle owl liver would reflect a decreasing trend in environmental exposure following the large-scale phase-out of lead in petrol. Besides lead and stable lead isotopes, we report liver concentrations of 14 other trace metals. To our knowledge, this is the first published study of long-term temporal changes in concentrations of lead and other trace metals in this apex nocturnal raptor.

## Materials and Methods

### Sample Composition and Distributions

Eagle owls found dead in Sweden belong to the state and must be reported and handed into the authorities for postmortem investigations. Tissue samples banked in the National Specimen Bank at the Swedish Museum of Natural History (SMNH) are stored at − 25 °C. Banked liver samples from a total of 122 dead eagle owls spanning from 1978 to 2013 were selected for this study. Only samples from wild-bred birds were chosen; released birds from captive breeding programs (Broo 1978; Hägerroth 1986)—all ringed and possible to identify—were excluded from the study. The selection included 59 males, 57 females, and 6 specimens of unknown sex. The temporal distribution of samples is summarized in Table 1. Assessments on cause of death were based on evidence from field-site circumstances and observations from necropsies conducted and reported by the National Veterinary Institute/SVA and by staff at SMNH. Data on weight were available for 54 males and 55 females. Judgements on body condition based on nutritional status were available in the database for 85 specimens filed into four categories: very good, good, lean, emaciated. Another 23 specimens were placed into these categories based on body mass as a proxy for condition. One outlier was excluded, and another 13 birds lacked data on weight or sex and could not be classified for condition. In some cases where death cause was recorded as “unknown” in the archive files, weight was used as a proxy for body condition to indicate for emaciation (starvation): a specimen with a weight less than two-thirds of the recorded mean weight for its gender in our sample was classified as starved. Because the mean weight for each gender was based on all sampled birds (including starved), it should be on the low side compared with if being based on live birds (for which data on weights were not attainable). The distribution among death causes is given in Table 1. The most frequent death causes were collisions with traffic and electrocutions. In another nine cases of collisions with electricity lines, it was not clear if the bird died of electric shock or of impact from the collision itself. Four of the 14 emaciated birds had also collided (3 with traffic, 1 with line).

**Table 1:** Temporal distribution of eagle owl specimens in the collection available for this study and their recorded cause of death

Time period	Male	Female	Male or female	All
1978–1984	5	11	1	17
1985–1989	10	5	0	15
1990–1994	7	11	1	19
1995–1999	14	7	1	22
2000–2004	10	8	1	19
2005–2009	3	7	2	12
2010–2013	10	8	0	18
All	59	57	6	122
*Cause of death*				
Collision traffic	11	15	5	31
Collision building	1	0	0	1
Collision lines	5	5	0	10
Collision lines/electrocution	5	4	0	9
Electrocution	14	14	0	28
Starvation	9	5	0	14
Starvation/Pb poisoning	0	1	0	1
Drowning	1	2	0	3
Illegal killing	0	2	0	2
Other trauma	0	2	0	2
Unknown	13	7	1	21
All	59	57	6	122

### Preparation of Samples and Analyses

Approximately 1.5 g of frozen liver tissue from 122 eagle owl specimens in the National Specimen Bank were subsampled and transferred to preweighed and acid-washed plastic capsules. Dry matter percentage was determined by weighing (± 0.1 mg) each sample before and after freeze drying. Freeze-dried and homogenized liver samples were digested with distilled nitric acid and hydrogen peroxide (10:1) in a microwave (CEM MARS 5) according to standard method SS-EN 13805. Blanks and certified reference materials 1577a (bovine liver, NBS) and DOLT-2 (dogfish liver, NRC-CNRC), as well as an internally certified control (fish liver, QM-LE), were included as quality controls in each digestion run.

After dilution of the digested liver samples with Milli-Q water, trace metal concentrations were determined with inductively coupled plasma mass spectrometry (ICP-MS; Thermo Scientific XSeries II) according to standard method SS-EN ISO 17294-2, using rhenium as internal standard. Lead concentrations were estimated as the sum of ^206^Pb, ^207^Pb, and ^208^Pb with measuring time set to 20 ms at each isotope with 50 sweeps and 3 replicates. The elements arsenic (As), chrome (Cr), nickel (Ni), and selenium (Se) were detected in CCT mode using H_2_ + He as collision cell gas, whereas lead (Pb), cadmium (Cd), copper (Cu), zinc (Zn), cobalt (Co), molybdenum (Mo), aluminum (Al), antimony (Sb), silver (Ag), bismuth (Bi), and tin (Sn) were measured in standard mode. Blanks were subtracted from the sample readings. Recoveries based on measurements of certified reference material are summarized in Table 2.

**Table 2:** Mean, median, and range of trace metal concentrations and stable lead isotope ratios in eagle owl liver (*n* = 122) measured by ICP-MS after microwave assisted digestion, along with levels of quantification (LOQ), number of records below LOQ, and mean recovery and precision based on certified reference materials (DOLT-2 and NIST-981)

Element	Liver concentrations (µg g^−1^ dw)				Level of quantification		Recovery (%)
	Mean	Median	Min	Max	LOQ	*n* < LOQ	DOLT-2 (*n* = 5)
Pb	0.179	0.103	0.015	1.55 (40.7)	0.012	0	92 ± 12%
Cd	0.197	0.165	0.009	0.89	0.0045	0	97 ± 1%
Cu	21.2	15.2	7.1	73.8 (172)	0.3	0	106 ± 3%
Zn	138	103	39	517	1.5	0	103 ± 5%
Se	4.34	3.61	0.72	19.2	0.15	0	99 ± 2%
Co	0.133	0.120	0.029	0.287	0.0015	0	102 ± 31%
Mo	2.49	2.44	0.57	4.46	0.0012	0	–
Al	8.9	3.5	*0.4*	83 (499)	1.5	25	80 ± 2%
Sb	*0.007*	*0.003*	*0*	0.21	0.12	66	–
Ag	0.007	*0.002*	*0*	0.10 (0.21)	0.005	83	104 ± 9%
Bi	*0.001*	*0.001*	0	0.008 (0.026)	0.0015	84	–
As	*0.10*	*0.08*	*0.03*	0.49 (1.24)	0.15	99	101 ± 4%
Cr	*0.016*	*0.01*	*0*	0.116 (0.265)	0.05	111	82 ± 22%
Ni	*0.015*	*0.01*	*0*	0.245 (2.015)	0.05	113	94 ± 17%
Sn	*0.006*	*0.002*	*0*	0.068	0.05	119	128 ± 4%

Isotope ratio	Mean	Median	Min	Max	NIST-981		NIST-981
					Measured ratio (*n* = 21)		Certified ratio
^206^Pb/^207^Pb	1.162	1.157	1.051	1.546	1.0860 ± 0.0072		1.0933 ± 0.0003
^208^Pb/^207^Pb	2.425	2.426	2.322	2.523	2.3758 ± 0.0140		2.3704 ± 0.0005

Calculations of means and medians are based on all measured concentrations without substitution for values below LOQ, except for a few extreme outliers (showed as Max concentrations within parentheses). Concentrations below LOQ in italic

Stable lead isotope ratios (^206^Pb/^207^Pb and ^208^Pb/^207^Pb) were estimated with ICP-MS in the same digested samples as for total lead but in a separate run with optimized settings (Measuring time 50 ms at ^206^Pb and ^207^Pb and 25 ms at ^208^Pb with 100 sweeps and 5 replicates). A dilution series of a certified reference solution (NIST-981 from National Institute of Standards and Technology, USA) indicated no significant relationship between Pb isotope composition and Pb concentration. The accuracy was good for ^208^Pb/^207^Pb and somewhat low for ^206^Pb/^207^Pb (Table 2). The relative difference between measured and certified isotope ratios was used to correct calculated isotope ratios for mass bias.

To minimize contamination, sample preparation was as much as possible performed in laminar airflow benches inside a clean room with positive air pressure and HEPA-filtered incoming air. Sample preparation and analyses followed the quality control routines of the laboratory’s accreditation.

### Statistical Analysis

The trace metal concentrations were skewed right and therefore log-transformed before the statistical analyses. There are a number of potentially confounding factors to consider in analyses of data on concentrations and trends of contaminants in wildlife (Peakall and Burger 2003). To study the relation between trace metal concentration and potential confounding factors including year, month of death (since the seasonal variation of Pb-concentrations is best described by a second-degree polynomial function, also the squared month was included in the multiple regression model), sex, total weight, longitude and latitude, multiple regression analyses were performed. To minimize the effect of significant confounding factors on interpretations of trends or spatial distributions, multiple regression also was used to adjust metal concentrations to the mean values of confounding factors, i.e., as if these factors were constant at their mean values. Possible temporal trends in trace metal concentrations were checked for significance using ordinary least square log-linear regression analysis. Also, the regression analyses were compared with the nonlinear Mann–Kendall trend test to check whether potential extreme values had a noticeable effect on the trend. Statistical power analysis was performed and the effect size, i.e., the lowest detectable trends at a power of 80% were estimated for the analyzed trace metals. The regression analyses and power calculations were performed before and after exclusion of extreme values. Extremes were identified using Tukey’s outer fence (Foreman 2014) within a gliding window of 5 years, but using 6 × IQR (Inter Quartile Range) instead of the original suggested 3 × IQR to get the filter more conservative. Differences between groups were tested with Mann–Whitney *U* test. A significance level of 5% was chosen for all tests. Ordinary Least Square and Multiple regression analyses were performed using the software package PIA (Bignert 2013).

## Results

Analytical performance and concentrations of all investigated metals and isotope ratios are summarized in Table 2. More than half of the measured concentrations were below the level of quantification (LOQ) for Sb, Ag, Bi, As, Cr, Ni, and Sn. The analytical recovery ranged between 92 and 106% except for Al and Cr that showed lower recoveries (80–82%) and Sn with 128% recovery. The precision of lead isotope ratios determined with single-quadrupole ICP-MS was adequate for our aim of estimating long term trends.

### Concentrations, Temporal Trends, and Relationships with Confounding Factors

**Fig. 1:**
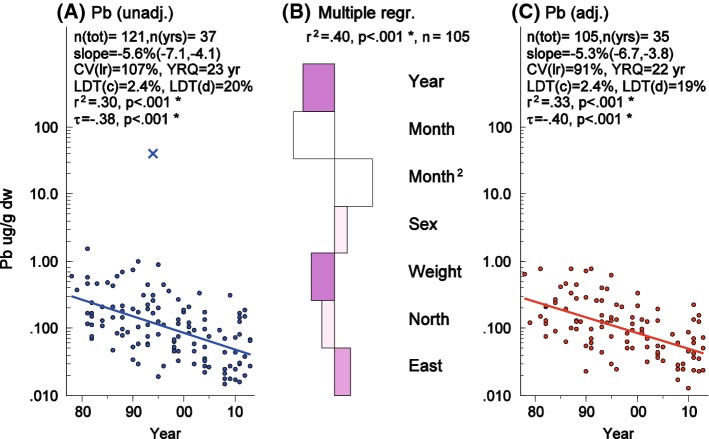
Lead (Pb) in eagle owl liver 1978–2013. One extreme specimen with 40.7 μg g^−1^ (left panel) is excluded from the loglinear trend estimations. CV is the Coefficient of Variation for the residuals along the regression line, YRQ is the number of years required to detect a 5% yearly trend with a power of 80%, LDT(c) is the Lowest Detectable Trend, expressed as a yearly change in percent, with an 80% power and using the sample size and number of years used in the current time-series, LDT(d) is the same but for a fixed period of 10 years, *r*^2^ is the Coefficient of Determination, τ is the Mann–Kendall’s tau. **a** Unadjusted Pb concentrations over time. **b** Standardized beta bar plot, the size of the bars is relative to the standardized beta-coefficients from a multiple regression analysis used to adjust the measured Pb-concentrations as if the potential confounding variables included in the regression models were constant at their mean values. Bars directed to the left are negative and to the right are positive. For sex, a bar directed to the right means higher concentrations in females. The color indicates *p* value: pale *p* < 0.05, medium *p* < 0.01, and dark *p* < 0.001. **c** Adjusted Pb concentrations over time calculated as if weight, sex, longitude, and latitude were constant

Lead concentrations in eagle owl liver (*n* = 121) ranged over two orders of magnitude (0.015–1.551 μg g^−1^ dry weight) with an overall mean of 0.179 μg g^−1^dw, median 0.103 μg g^−1^ dw (Table 2) and with 95% of the samples < 0.600 μg g^−1^. This mean, median, and range excludes one individual that showed an extreme concentration of 40.7 μg g^−1^ dw. Lead concentrations were significantly correlated with total weight (*p *< 0.001), sex (*p *< 0.05), longitude (*p *<0.01), and latitude (*p *< 0.05; Fig. 1b) but not with % dry matter in the liver samples. Lead concentrations were adjusted for sex, total weight, latitude, and longitude before temporal trend estimation and for year, total weight, and sex before estimating spatial distribution. Using adjusted concentrations accordingly decreases the residual variances around the regression line for temporal changes and hence increases the statistical power to detect trends and decreases the required effect size (Fig. 1a, c).

The percentage of dry matter in the liver samples showed a small but significant decrease over the study period: 0.27% per year before adjusted, and 0.35% adjusted for weight, sex, and increasing values to the north. However, the correlation between measured concentration and percentage dry matter in livers is not significant for lead or any of the other analyzed trace metals. When the metal concentrations are adjusted for other confounding factors, there is no need for further correction of dry matter. The mean value for content of dry matter is 27.2% (range 18.9–32.6).

Correlations with confounders, observed temporal trends and estimated effect size for all studied trace metals are summarized in Table 3. Before adjustment for confounders and exclusion of extreme values a negative temporal trend was observed for Pb, Zn, Al, Ag, and As, but after adjustment this remained only for Pb and As. Exclusion of extremes resulted in a significant negative trend also for Sb.

**Table 3:** Relationships between trace metal concentrations and potential confounding variables: body weight, latitude, longitude, and sex

Element	Confounding factors								Time trends (% yr^−1^)				
	Body weight		Latitude		Longitude		Sex		Trend	*p*	Effect size %		Excl.
	Sign	*p*	Sign	*p*	Sign	*p*	Sign	*p*			35 yr	10 yr	extr.
Pb	–	< 0.001	–	< 0.05	+	< 0.01	+	< 0.05	− 5.3	< 0.001	2.4	19	1
Cd		ns		ns		ns		ns		ns	3.2	24	0
Cu	–	< 0.001		ns		ns	+	< 0.001		ns	1.4	8.7	1
Zn	–	< 0.001		ns		ns	+	< 0.001		ns	1.0	6.8	3
Se	–	< 0.01	–	< 0.01	+	< 0.01		ns		ns	0.95	5.9	4
Co	–	< 0.001	–	< 0.01	+	< 0.01	+	< 0.001		ns	1.1	6.9	0
Mo	–	< 0.001		ns		ns		ns		ns	0.74	4.8	0
Al	–	< 0.01		ns		ns	+	< 0.05		ns	3.3	24	10
Sb		ns		ns		ns		ns	− 2.0	< 0.05	3.4	25	3
Ag	–	< 0.001		ns		ns		ns		ns	3.6	28	4
Bi		ns		ns		ns		ns		ns	2.9	21	2
As	–	< 0.01		ns		ns	+	< 0.05	− 1.5	< 0.01	2.0	12	4

Sign: implies a negative correlation and + implies a positive correlation between metal concentration and an increase in weight, latitude, longitude and sex (higher in females), with all other co-variables kept constant. Effect size % is calculated as the Lowest Detectable Trends at 80% power for the available sample spanning more than 35 years or as if the sample would comprise only 10 years of data. Excl. extr. = number of excluded extremes using an outer fence based on 6 × IQR. Trends for chrome, nickel, and tin were not estimated due to few observations > LOQ (e.g., Table 2)

A significant decrease in eagle owl liver Pb concentrations occurred during the study period (*p *< 0.001; Fig. 1). Based on the log-linear time-trend curve and excluding one extreme value of 40.7 µg g^−1^ (dw), a mean annual decrease rate over the 36 years was 5.6% and 5.3% when adjusting for confounding factors.

**Fig. 2:**
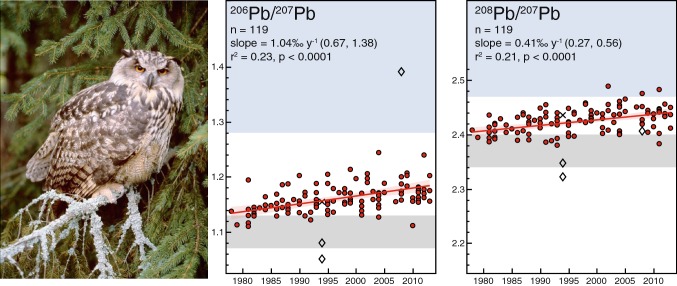
Stable Pb isotope ratios (^206^Pb/^207^Pb and ^208^Pb/^207^Pb) in 122 eagle owl liver samples show increasing trends 1978–2013. Diamonds mark three extreme ratios that are excluded from trend estimates; X marks a specimen with an extreme total Pb concentration of 40.7 µg g^−1^. Grey ranges indicate isotope ratios of European leaded gasoline (from Hansmann and Köppel 2000), and blue indicates lower range of the Baltic-Shield isotopic background derived from soils (Reimann et al. 2012) and lake sediments (Renberg et al. 2002). Eagle owl photo by Viking Olsson

In parallel with the decrease in owl-liver lead concentration over the study period, the isotope ratios ^206^Pb/^207^Pb and ^208^Pb/^207^Pb increased linearly (*p *< 0.0001) with on average 0.0012 (or 0.10% if log-transformed) and 0.0010 (0.04%) per year, respectively (Fig. 2). Average ^206^Pb/^207^Pb increased from 1.138 in 1978–1985 (*n* = 18) to 1.170 in 2010–2013 (*n* = 18), whereas ^208^Pb/^207^Pb increased from 2.408 to 2.435.

The effect size, as reported in Table 3, is especially important when the outcome from the regression analysis is not significant. It can be interpreted as the magnitude of a trend that would most likely (with an 80% chance) be detected in case it was present, e.g., for Cd no trend was detected and most likely there is no true trend, at least not larger than 3.2% per year. An increased sample size, longer time period or decreased variability would decrease the effect size and hence make the time-series more efficient to detect true trends. The effect size is thus an indirect measure of the variability in the metal analyses. The essential trace metals Cu, Zn, Se, Co, and Mo show a lower variability than the nonessential elements (Table 3). It should be noted that a trend lower than the effect size may be significant in a single data set (Sb, As), but the chance to detect this trend in all possible trials, with samples of the same size and variance, from the same population, would be less than 80%, i.e., the statistical power will be less than 80%.

Comparison of effect sizes between different scenarios, as in Table 3, illustrates the benefits of long time series for trend estimates: a 10-year sample would have been useless for a trend study of nonessential elements, and almost so also for the essential metals.

### Spatial Distributions

**Fig. 3:**
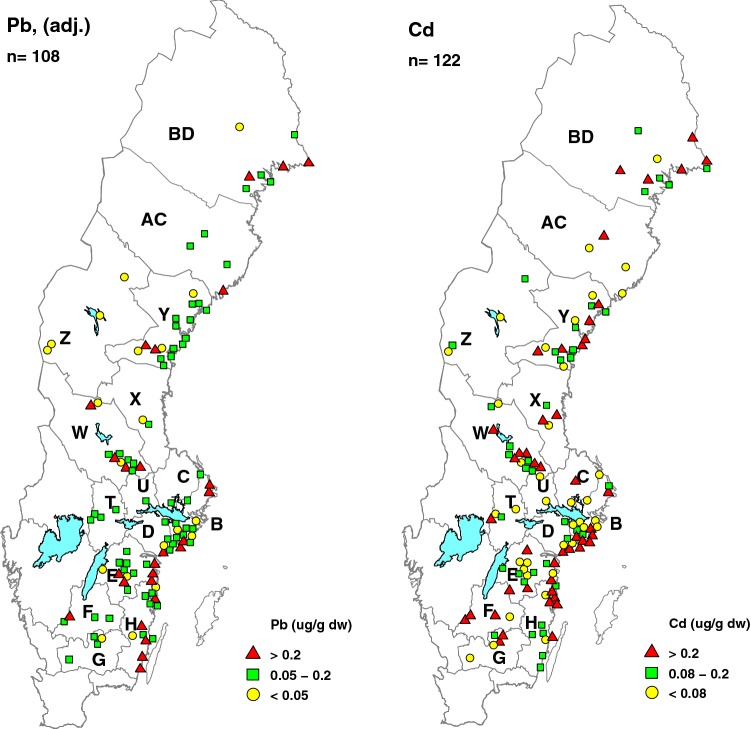
Distribution of lead (Pb) concentrations adjusted for weight, temporal trend and sex (*n* = 108) and cadmium (Cd) concentrations (unadjusted, *n* = 122) in liver from eagle owl in Sweden

The geographical distribution of eagle owl samples is shown in Fig. 3, with a division into concentration intervals for lead and cadmium. There are more eagle owls with lead concentrations in the higher intervals toward the eastern, coastal areas, and a tendency for more birds with lower concentrations toward the north (Fig. 3 Pb). For cadmium (Fig. 3 Cd) the concentrations are fairly evenly distributed among the three intervals, and there is no clear tendency for higher concentrations toward the east, or lower toward the north, except for only low concentrations in the few western birds from the province of Jämtland (Z) in northern Sweden.

### Seasonal Variation of Body Mass, Condition, and Lead Concentrations

**Fig. 4:**
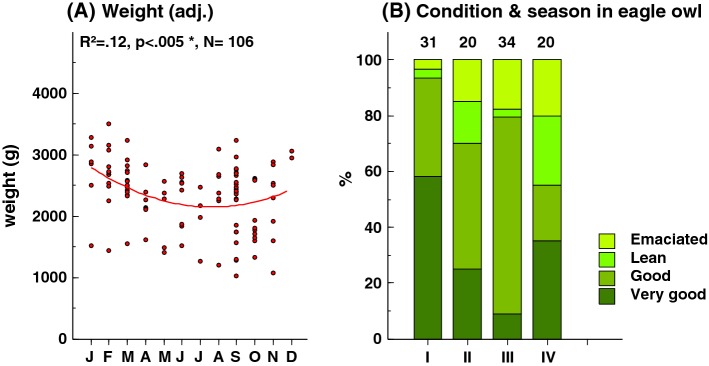
Seasonal variation in **a** body weight adjusted for year, sex, and **b** body condition of eagle owls in Sweden. The distribution of body condition in four classes are grouped into four time periods: *I* January–March, *II* April–June, *III* July–September, *IV* October–December. Class sample sizes in upper row in b. One Pb-poisoned bird excluded

The average body weight is clearly higher in females compared with males (Table 4). Body mass confounds the measured concentrations of lead in eagle owl liver so that heavier individuals of each sex show lower lead concentrations at similar environmental lead exposure (Fig. 1). Weight also shows a significant seasonal variation and was higher during winter (Fig. 4a). These factors complicate the interpretation of lead concentrations in eagle owl over time and have been corrected for when statistically significant.

**Table 4:** Body mass (g) and liver Pb concentrations (μg g^−1^ dw) among male and female eagle owl found dead in different periods of the year

Period	Male			Female		
	*N*	Mean	Range	*N*	Mean	Range
*Body weight* (g)						
All year	53	1993	858–2932	55	2670	1434–3929
Jan–Mar	11	2381	2064–2932	21	2942	1720–3929
Apr–Jun	13	1836	1183–2389	7	2550	1700–3060
Jul–Sep	18	1981	858–2786	16	2473	1434–3460
Oct–Dec	11	1772	1193–2783	11	2511	1540–2724
*Pb* (µg g^−1^ dw)						
All year	58	0.083	0.019–0.887	54	0.107	0.016–1.551
Jan–Mar	12	0.074	0.031–0.217	22	0.078^a^	0.016–1.009^a^
Apr–Jun	13	0.058	0.020–0.364	7	0.120	0.046–1.551
Jul–Sep	20	0.091	0.019–0.887	16	0.112	0.046–0.692
Oct–Dec	13	0.168	0.022–0.608	9	0.147	0.025–0.732

^a^One extreme excluded

Seasonal distributions in body weight for male and female eagle owls are summarized in Table 4. The body mass was higher during January-March compared with the rest of the year for both sexes. The seasonal variation in body weight is inherently mirrored in the seasonal distributions in body condition (Fig. 4b) with most birds in very good condition in January–March, lower proportions in the spring and summer periods and higher again in the autumn period.

There is no apparent seasonal difference in Pb concentrations in the material (Table 4). Both median and the lower boundary for range of liver Pb concentrations were twice as high in females compared with males during April through June, but the difference is not statistically significant (*p* = 0.18).

**Fig. 5:**
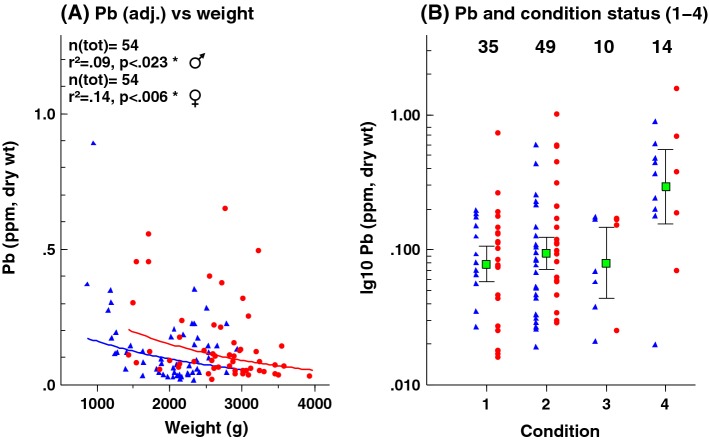
Pb concentrations (μg g^−1^ dw) in **a** male and female eagle owls grouped on basis of body weight (g) and **b** birds classified on basis of body condition: 1 = very good, 2 = good, 3 = lean, 4 = emaciated; blue triangles = males, red circles = females. Class sample sizes in upper row in **b**, N_tot_ = 108 (one emaciated, Pb-poisoned bird excluded)

Figure 5 shows the distribution of Pb concentrations in male and female eagle owls grouped on basis of body mass (a) and in specimens classified after body condition (b). A significant log-linear relationship between weight and Pb concentration exists for both male and female eagle owls, and birds classified as emaciated (Fig. 5b) show significantly higher concentrations (*p *< 0.001).

## Discussion

### Sex, Body Weight, and Condition

A significant relationship was found between body weight in eagle owls and the concentrations in liver of Pb (Fig. 4), Cu, Zn, Se, Co, Mo, Al, Ag, and As, with higher levels in lighter, possibly starved specimens (Table 3). For lead, emaciated birds had significantly higher concentrations (Fig. 5b). In the same way, concentrations of heavy metals increased in liver as nutrient reserves fell in common buzzards *Buteo buteo* in the Netherlands (Jager et al. 1996), whereas Pb concentrations in liver showed no clear relationship with body condition in barn owls *Tyto alba guttata* in the same country (Esselink et al. 1995). Observations of differences like these between sympatric species call for attention to host factors that need to be considered when interpreting measured concentrations.

Eurasian eagle owls show pronounced sexual dimorphism: in our sample (excluding emaciated specimens) females weighed on average 30% more than males. Both male and female eagle owls were heavier during January through March than during the rest of the year (Table 4). In live-trapped snowy owls *Bubo scandiacus* in Canada, females but not males tended to put on fat during the winter months, and in dead birds males had less fat reserves and a higher incidence of starvation and disease than females (Chang and Wiebe 2016). In our material collected in the field year-round, 9 of 53 males were emaciated, but none of the 9 starved males were found during the winter months (January through March). Excluding all emaciated individuals, male and female weights, respectively, averaged 14.7% and 16.3% higher in January-March compared with the rest of the year. This similarity in higher weights in males and females during winter indicate that food limitation and competition for food between the sexes was low and that eagle owls were capable of building up in condition during winter, a favorable trait for potentially improved reproductive output and fitness. A tendency for slightly lower Pb levels in the winter samples (Table 4) is consistent with the significant relationship that was found with body weight.

Female eagle owls showed significantly higher concentrations of Pb, Cu, Zn, Co, and As than males (Table 3). Higher liver levels of Pb, Zn, and As in females than in males also has been reported in waterfowl (Taggart et al. 2006, Lucia et al. 2010). There was no apparent difference in Pb levels between male and female eagle owls in summer, autumn, and winter, whereas in spring (April–June) the observed median and lower boundary of the range were twice as high in females as in males (Table 4). Although not statistically significant, the observation is noteworthy. For breeding adults, this period includes the time of egg production, incubation, and most of the nestling period that may exert different physiological strains in males and females. This could influence the residue concentrations, but information on breeding was not available among birds in our material. Significantly higher accumulation of Pb in bone tissue of females compared to males has been observed in experimental studies with Pb-dosed mallard ducks (Finley et al. 1976), apparently a result of calcium mobilization from bone during eggshell formation (Finley and Dieter 1978). In a controlled dose–response study with breeding American kestrels, the females accumulated significantly higher liver Pb concentrations than the males (Pattee 1984).

### Thresholds and Trends

Although the observed negative trend for % dry matter in the liver samples was weak, the result is surprising; if a change would be anticipated, it would rather be in the opposite direction if freeze-drying during storage had occurred. Furthermore, a decrease in dry matter content was significantly correlated with an increase in body weight. A possible explanation for this could be if the lipid content in liver would decrease with an increase in body weight, but we found no evidence for this in literature.

Concentrations increased significantly towards east and decreased towards north for Pb, Se, and Cu (Table 3). Relatively higher concentrations could reflect the generally stronger exposure to anthropogenic sources close to the coast and towards the south. Differences in prey composition will be reflected in the residue levels, e.g., eagle owls inhabiting coastal habitats preying on waterfowl would be exposed to higher concentrations than owls feeding mainly on voles in inland rural areas. Individual preferences in habitat use also will have an effect: a study of radio-tracked ring-billed gulls (*Larus delawarensis*) from the same breeding colony showed that the time spent foraging in landfills and wastewater basins differed and correlated positively with liver Pb concentrations (Brown et al. 2018). Changes in habitats over time also may cause switches in prey availability (Penteriani et al. 2002). However, our material collected ad hoc all over Sweden during a 36-year study period give no way to account for effects of possible changes in prey composition and differences in prey utilization. Monitoring of Pb concentrations in moss *Pleurozium schreberi* and *Hylocomium splendens* in Sweden showed a highly significant decrease 1975–2015 and a spatial pattern with clearly lower levels to the north and northwest and locally higher levels on the eastern coast (Danielsson and Pihl Karlsson 2016). However, the more frequent occurrence of eagle owls with higher Pb levels on the southeastern coast (Fig. 3) is not reflected in concentrations in moss, suggesting an influence from coastal prey in these owls.

None of the eagle owls in this study were reported to have died because of lead poisoning (Table 1), but one specimen had a concentration (40 μg g^−1^) that far exceeds a generally accepted threshold of around 20 μg g^−1^ dw in liver for clinical poisoning in birds (Pain et al. 1995; Franson and Pain 2011). The necropsy death-cause filed for this specimen was emaciation, a common feature observed in victims from secondary lead poisoning caused by ingestion of metallic lead (Samour 2016; Krone 2018). Scavengers are exposed to lead from spent ammunition in discarded meat or in carcasses. Another source of metallic lead, potentially of more concern for the eagle owl that is generally not a scavenger, would be gunshot in wounded free-ranging birds and small mammals. Such animals would fare a greater risk to be predated. A study on the Bonnelli’s eagle *(Aquila fasciata)* in Spain showed a high occurrence of lead shot in the eagle castings and indicated that injured small game was the main source of lead contamination in this non-scavenging raptor (Gil-Sánchez et al. 2018). Incidences of Pb poisoning in eagle owl have been reported from Spain (Mateo et al. 2003) and Korea (Kim and Oh 2012). The individual here with the extreme concentration also had the highest concentration of antimony (Sb), a metal often used in alloys with Pb, e.g., in lead shot, which may indicate that this bird had been poisoned by ingesting ammunition. However, Pain et al. (1992) did not find an efficient uptake of Sb from lead shot ingested by water birds. The low frequency of Pb poisoning in the present study indicates that ingestion of spent ammunition has been rare in eagle owl in Sweden. Beside the one Pb poisoned specimen, all the eagle owls in this study had liver Pb concentrations that fall below a threshold for subclinical poisoning suggested by Franson and Pain (2011) and probably represent mainly background concentrations.

A mean annual decrease in total Pb of 5.6% before and 5.3% after adjustment for confounding factors occurred over the study period. Similar mean annual decrease rates for liver Pb concentrations in terrestrial wildlife in Sweden have been reported by Lind et al. (2006) for moose *Alces alces* (8.8% 1980–2003) and reindeer *Rangifer tarandus* (3.5% 1983–2003) and for kidney in starling *Sturnus vulgaris* in the range 6.2–12% in different areas 1982–1999. Monitoring of metal concentrations in fish liver in Sweden also have shown significant decreases for Pb in pike *Esox lucius* in lake Storvindeln (4.3% 1968-2013), herring *Clupea harengus* in the Baltic Sea (around 5% 1980–2013), and cod *Gadus morrhua* (5.1% in the Baltic Sea and 2.5% in the Kattegat 1983–2013; Bignert et al. 2014). For the eagle owl samples in this study, the total reduction over the study period was 87% (Fig. 1). Bustnes et al. (2013) reported a similarly high decrease of 95% for lead in feathers from tawny owl (*Strix aluco*) in Norway for the time period 1986–2005. Lind (2012) reported liver Pb concentrations of up to 0.076 µg g^−1^ on a wet weight basis in Swedish eagle owls from around year 2000. Based on a mean percentage dry matter of 27%, as found in the current study, this would correspond to a concentration of 0.28 µg g^−1^ dw, which is in agreement with the highest concentrations around year 2000 in Fig. 1.

The widespread decrease in Pb concentrations in biota over the past decades has generally been attributed to the phase out of leaded gasoline. In Europe, addition of Pb to gasoline has been regulated stepwise over many years: in Sweden starting in 1970 and with a full ban on Pb in car petrol enforced in 1986, followed by a total ban on Pb in all kinds of motor fuel in 1995. A study on environmental Pb in kestrel *Falco tinnunculus* liver before (1995–1997) and after (2001) restrictions were placed on leaded fuel in Spain showed a significant decrease in concentrations in birds from rural and city sites even over this short time period, but not so in birds from a former mining site (García-Fernández et al. 2005). The mean value and lower boundary for range of Pb in eagle owl liver reported from Spain (García-Fernández et al. 1997) were about three times higher (compared on dw basis) than in this study. The difference may be attributed to the later enforcement of leaded fuel regulations in Spain, but regional contamination in the area from which the Spanish eagle owls originated also may be important. The region has a long mining history and a dry climate facilitating wind dispersal of lead from exposed mine tailings (Brotons et al. 2010).

The observed increase in the isotope ratios ^206^Pb/^207^Pb and ^208^Pb/^207^Pb supports that the decrease of lead concentrations in biota, including eagle owls, is linked to declining atmospheric lead pollution, especially that from leaded gasoline combustion. Background Pb isotope composition in Precambrian rock that dominates the Baltic Shield is considerably more radiogenic, i.e. with higher ^206^Pb/^207^Pb, than the earth crust at large and especially than the lead ores that were used for gasoline additives in Western Europe (Fig. 2; Komárek et al. 2008). Increasing isotope ratios towards the background levels is thus expected as the influence from this modern pollution source is diminishing. However, the increase is very slow compared with the comparably rapid decrease in total lead, reflecting the long history and global reach of anthropogenic Pb emissions to the atmosphere (Bindler 2011), while the weathering of granite bedrocks is a very slow process. Nevertheless, it is important to account for this significant temporal change when comparing stable Pb isotope ratios in samples from different periods.

Two individuals showed much lower Pb isotope ratios than the others. These were both found dead on two different mining sites in the County of Dalarna (W). Very low ^206^Pb/^207^Pb (1.024) and ^208^Pb/^207^Pb (2.297) have been reported for the sulfide ore in this area (Sundblad 1994). Eagle owls are known to be strongly territorial throughout their adult life and it is possible that the isotopic composition of individuals nesting and hunting on mining ground is influenced by exposure to lead leaking from the tailings that have accumulated over centuries in this area.

Among other trace elements than lead: Cadmium concentrations ranged between 0.01 and 0.9 µg g^−1^ dw, which is far below a suggested critical threshold of 45–70 µg g^−1^ wet weight (≈ 150–210 on dw basis) (Wayland and Scheuhammer 2011). Levels of Se ranged between 0.72 and 19.2 µg g^−1^ with a median at 3.6 µg g^−1^. Selenium concentrations < 10 µg g^−1^ have been considered as “background” for terrestrial species but levels in the range 10–20 µg g^−1^ as potentially toxic (Ohlendorff and Heinz 2011). The upper range Se level in our sample should therefore be of concern. Arsenic levels in our study (Table 2) were below the mean levels of 3–6 µg g^−1^ found in four owl species in Spain (Perez-Lopez et al. 2008) and far below a suggested deleterious level of 56 µg g^−1^ measured in a dead North American osprey (Wiemeyer et al. 1980). Zinc levels in the four owl species in Spain (Perez-Lopez et al. 2008) averaged 2–3 times higher than in this study, but the highest concentrations in our sample (Table 2) were within the lower ends of ranges for levels associated with deleterious effects in waterfowl (Doneley 1992; Levengood et al. 1999; Sileo et al. 2003). For other trace metals studied here, there is not much published information about critical thresholds for concentrations in bird liver. Mean concentrations of As, Cd, Pb, Cu, and Zn in eagle owl liver in this study were of the same magnitude as in liver of nestling Tengmalm’s owl *Aegolius funereus* 1984–1985 from the county of Västerbotten (AC, Fig. 3) in northern Sweden (Hörnfeldt and Nyholm 1996).

## Conclusions

In accordance with our hypothesis, lead concentrations in eagle owl in Sweden have decreased at a similar rate as reported for other biota in Sweden during the study period. The temporal patterns of stable isotope ratios ^206^Pb/^207^Pb and ^208^Pb/^207^Pb in eagle owl liver indicate that the decrease of lead concentrations in eagle owls, as well as in other biota is a positive result from the regulations of use of lead as additive to motor fuels. A single suspected incidence of lead poisoning among 122 specimens indicates that ingestion of metallic lead is rare in eagle owl in Sweden, in contrast to what has been reported for white-tailed sea eagles (Helander et al. 2009) and golden eagles *Aquila chrysaetos* (Ecke et al. 2017). As for other trace elements, the concentrations were with very few exceptions low and our results indicate that poisoning by the metals in this study were rare and did not pose a threat to eagle owls in Sweden during the study period.
